# EQUIP emergency: can interventions to reduce racism, discrimination and stigma in EDs improve outcomes?

**DOI:** 10.1186/s12913-022-08475-4

**Published:** 2022-09-02

**Authors:** Colleen Varcoe, Annette J. Browne, Nancy Perrin, Erin Wilson, Vicky Bungay, David Byres, Nadine Wathen, Cheyanne Stones, Catherine Liao, Elder Roberta Price

**Affiliations:** 1 Critical Research in Health and Healthcare Inequities Research Unit, School of Nursing, The University of British Columbia, Vancouver, BC Canada; 2 Johns Hopkins University School of Nursing, Baltimore, MD USA; 3 School of Nursing, University of Northern British Columbia, Prince George, BC Canada; 4 Provincial Health Services Authority, Vancouver, BC Canada; 5 Arthur Labatt Family School of Nursing, Western University, London, ON Canada

**Keywords:** Health inequity, Health disparities, Stigma, Discrimination, Racism, Emergency, Intervention research, Health equity, Emergency departments, Indigenous

## Abstract

**Background:**

Despite a publicly funded system, health care in Canada has been shown to be deeply inequitable, particularly toward Indigenous people. Based on research identifying key dimensions of equity-oriented health care as being cultural safety, harm reduction and trauma- and violence-informed care, an intervention to promote equity at the organizational level was tested in primary health care, refined and adapted, and tested in Emergency Departments (EDs).

**Methods:**

In partnership with clinical, community and Indigenous leaders in three diverse EDs in one Canadian province, we supported direct care staff to tailor and implement the intervention. Intervention activities varied in type and intensity at each site. Survey data were collected pre- and post-intervention from every consecutive patient over age 18 presenting to the EDs (*n* = 4771) with 3315 completing post-visit questions in 4 waves at two sites and 3 waves (due to pandemic constraints) at the third. Administrative data were collected for 12 months pre- and 12 months post-intervention.

**Results:**

Throughout the study period, the participating EDs were dealing with a worsening epidemic of overdoses and deaths related to a toxic drug supply, and the COVID 19 pandemic curtailed both intervention activities and data collection. Despite these constraints, staff at two of the EDs mounted equity-oriented intervention strategies; the other site was experiencing continued, significant staff shortages and leadership changeover. Longitudinal analysis using multiple regression showed non-significant but encouraging trends in patient perceptions of quality of care and patient experiences of discrimination in the ED. Subgroup analysis showed that specific groups of patients experienced care in significantly different ways at each site. An interrupted time series of administrative data showed no significant change in staff sick time, but showed a significant decrease in the percentage of patients who left without care being completed at the site with the most robust intervention activities.

**Conclusions:**

The trends in patient perceptions and the significant decrease in the percentage of patients who left without care being completed suggest potential for impact. Realization of this potential will depend on readiness, commitment and resources at the organizational and systems levels.

**Trial registration:**

Clinical Trials.gov #NCT03369678 (registration date November 18, 2017).

## Background

Health care in Canada has been shown to be deeply inequitable, particularly toward Indigenous people [1–11]. Efforts to promote equity have been made at the national and international policy levels, and at the level of educating care providers [12–19]. Interventions to promote equity are often aimed at improving health care access and care for individuals and studied within the context of specific health issues such as diabetes [20], stroke [21], cancer screening [22], or organ transplant [23]. However, our research on health and health care inequities with Indigenous and non-Indigenous people pointed to the importance of intervention at the level of health care organizations [24–27]. This research identified the key dimensions of equity-oriented health care (EOHC) as requiring attention to cultural safety, harm reduction and trauma- and violence-informed care. Based on this work, with EOHC serving as the theoretical grounding, we designed an intervention to promote equity at the organizational level [25]. Our intention was to go beyond a focus on education of providers as an equity-promoting strategy that can imply holding them responsible for inequities and can fail to account for contextual constraints. We studied implementation of this intervention, “Equipping Health Care for Equity” (EQUIP) in four diverse primary health care (PHC) settings. This study (EQUIP PHC) demonstrated that EOHC was associated with significantly better patient self-reported health outcomes [28] and improved staff confidence and comfort in providing EOHC [29]. Despite these impacts, the intervention did not show any direct changes in self-reported patient outcomes, and our analysis suggested that the intervention needed to be more directly “owned” by direct care providers, offered more intensively over a shorter period of time, and could have been more radically disruptive in relation to counteracting the ongoing stigma that people experience, particularly for people with substance use issues [29, 30].

The findings of EQUIP PHC underscored the importance of Emergency Departments (EDs) promoting equity across the primary care continuum [28, 29, 31, 32]. Each of the PHC clinics identified that those they served often faced poor treatment when accessing care at local EDs. These findings aligned with historical and ongoing evidence of inequitable treatment in EDs for Indigenous people [4, 7, 33, 34], people experiencing violence [35, 36], and those presenting with histories of mental illness [37], substance use and/or homelessness [38, 39].

Research to date has not studied equity-oriented interventions in Emergency settings. However, the consequences of inequitable treatment of Indigenous people in EDs have been described [4, 7, 33, 34, 40], as have efforts to integrate cultural safety in EDs [41]. Building on such research, and the lessons learned in our primary health care research, we modified the intervention, tailored it to and tested it in EDs [42]. This study offers concrete direction for organizations to improve care and outcomes for people who are least well served in EDs, including, but not limited to, Indigenous people. An intersectional analysis of pre-intervention baseline data from this study, published elsewhere [43], showed some of the complex social circumstances associated with perceptions of poor care and discrimination in EDs. Specifically, those reporting the lowest perceptions of care were those most severely socially and economically disadvantaged, including a high proportion of Indigenous people.

## Methods

EQUIP Emergency is a study of an organizational-level intervention (in contrast to interventions aimed at individual service providers) to improve care quality at the point of care for those who face health inequities. This study is a three-way collaboration among health researchers, health care staff and Indigenous/community leaders aimed at developing an evidence-based organizational level intervention to promote equity for Indigenous and non-Indigenous people in diverse EDs. The study is part of a broader program of research entitled EQUIP Health Care that aims to reduce health inequities at the point of care in pursuit of the quadruple aims of health system optimization: improving the health of populations, enhancing patient experiences and outcomes, reducing the per capita cost of care, and improving the work life of staff [44], and aligns with more recent calls to add equity as the fifth aim [45, 46]. In partnership with clinical, community and Indigenous leaders in three diverse EDs in one Canadian province, we supported direct care staff to tailor and implement the intervention. The EDs are each in different health authorities and geographical settings, and included a) St. Paul’s Hospital (SPH) serving an urban area, b) Surrey Memorial Hospital (SMH) serving a large suburban area, and c) the University Hospital of Northern British Columbia, serving a region of rural, remote, and small urban communities, and relatively higher proportions of Indigenous peoples compared to other regions. The characteristics of each have been more fully described elsewhere [42].

Intervention activities varied in type and duration at each site. In brief, as outlined in the protocol [42], the intervention was built on our understanding of equity and EOHC informed by critical theoretical understandings of social justice, and the structures that perpetuate health and social inequities, and our previous research on interventions to promote EOHC. The overall study was guided by complexity theory using an integrated approach to implementing and mobilizing interventions. Complexity theory considers the diverse and complex interactions within systems [47–49]. The intervention was shaped by tenets of complexity theory, as EDs are complex adaptive systems with many interacting parts that influence one another. These approaches and our three-way leadership model were complemented by a change leadership approach known as Front Line Ownership (FLO) [50, 51], all of which align with understanding health care systems and EDs as complex adaptive systems. FLO aims to engender commitment beyond “buy in” and is based on the understanding that those closest to the process of care are best placed to identify and implement change [51]. We anticipated that working with organizational leaders to support direct-care staff to lead change would identify effective intervention strategies, and aiming at the organizational level would avoid “blaming” direct care staff for inequities. In each site, direct care staff were invited to information sessions, and invited to form working groups (WGs). These WGs were supported with paid time approved in advance by executive and management leadership, by a site-specific research assistant (provided by our research team) to help organize meetings, and a catalyst grant of $10,000 to support activities identified as priorities by the WGs. Each site also was provided with a) a workbook to guide organizational change processes, b) access to online learning modules to orient staff to the key dimensions of EOHC, and c) access to “change coaches” and “content experts” to work with as they wished, which were variously taken up to greater or lesser extents by each site.

The participating EDs were confronted with a dramatically worsening epidemic of deaths related to a toxic drug supply throughout the study period and the COVID 19 pandemic curtailed both intervention activities and data collection. Despite these constraints, staff at two of the EDs (SPH and SMH) were able to form a Working Group (WG) and mount equity-oriented intervention strategies; the other site (UHNBC) was experiencing significant and enduring staff shortages and leadership turnover [52]. Despite initially strong expressions of interest with regard to the intervention approaches, UHNBC did not form a working group or undertake any intervention activities during the intervention period. SMH formed a working group comprised primarily of ED nurses who undertook some intervention activities, including work to improve patient way-finding, and equity-oriented messaging in waiting room televisions; these activities were truncated by the COVID 19 pandemic, and the WG was suspended. SPH also formed a working group, again comprised primarily of ED nurses, and undertook activities that included improving signage at triage, installing TV monitors with equity-oriented and anti-stigma messages, and partnering with the hospital Indigenous health team and local Indigenous communities and an artist to commission and install artwork to create an improved patient environment in the waiting room. The SPH working group members remained engaged with one another throughout the COVID 19 pandemic to complete the aforementioned activities. It is of importance to note that during the intervention period at SPH, hospital-initiated staff training on substance use and stigma (“the Safe Care Program”) was concurrently implemented with specific attention to Indigenous-specific racism, and a 10-bed rapid access unit for people who use substances was opened to provide appropriate care specific to people with substance use related issues and relieve pressure on the ED.

A mixed methods multisite design was used to examine changes in key outcomes specified in the intervention theory [42]. We hypothesized increases in patients’ mean overall ratings of care and in staff perceptions of care (primary outcomes), and decreases in patient experiences of discrimination in the ED and increases in staff engagement and team effectiveness (secondary outcomes) (Tables 1 and 2).

**Table 1:** Patient survey measures

Concept	Instrument and Source	Items	Example Item	Range
Gender and sexual orientation	Rainbow Health Ontario [53]	2	NA	NA
Housing/living situation	Housing stability [54]	1	NA	NA
Difficulty living on income	Financial Strain Index [55]	1	NA	NA
Discrimination in Everyday Life	Everyday Discrimination Scale [56]	9	You are treated with less courtesy than other people are.	0–5 Overall score: 0–45
Discrimination during ED Visit	Discrimination in Medical Settings Scale [57]	7	You felt like a doctor or nurse was not listening to what you were saying.	1–5 Overall score: 7–35
Experiences of Care	Emergency Department Patient Experiences of Care (EDPEC) Scale [58]	15	Using any number from 0 to 10, where 0 is the worst care possible and 10 is the best care possible, what number would you use to rate your care during this emergency department visit?	Quality of Care: 0–10
	British Columbia EDPEC [59]	9	NA	NA
	Investigator developed (EQUIP ED)	12	During this visit, did staff make you feel welcome?	Yes/No Overall score: 0–12
Patient Acuity on Presentation	Canadian Triage Assessment Scale (CTAS) [60]	1	NA	1–5

**Table 2:** Staff survey measures

Concept	Instrument and Source	Items
Your work experiences	Accreditation Canada’s Worklife Pulse Tool [61]	25
Team effectiveness	Canadian Institute for Health Information’s PHC Team Effectiveness Scale [62]	11
Perceptions of patient care	Investigator developed (EQUIP ED)	11
Cultural safety	Investigator developed (EQUIP ED)	5
Trauma- and violence-informed care	Investigator developed (EQUIP ED)	5
Care related to substance use	Investigator developed (EQUIP ED)	7
Work experiences during COVID 19	Investigator developed (EQUIP ED)	11
Demographics	Rainbow Health Ontario [53]	1
	Investigator developed (EQUIP ED)	11

Data from hospital administrative sources were reviewed to determine which organizational-level variables could be hypothesized to be sensitive to EOHC, were comparable across all three sites and had sufficient stability over time. As shown in Table 3 variables included the number of people who left the ED without care completed (LWCC) (primary outcome) as a percentage of the total number of people seen, and the rate of staff sick time taken as a proportion of productive hours (primary outcome), as well as variables used for descriptive purposes.

**Table 3:** Administrative data variables collected monthly

Type	Variable
Patient data	CTAS Level 1–5
	Volume of patients
	Distribution by age group (19–100+)
	Sex
	Re-admission to emergency department within 30 days of discharge
	Flagged as homeless
	Left without care complete
Staff data	Productive hours
	Sick time

**Fig. 1:**
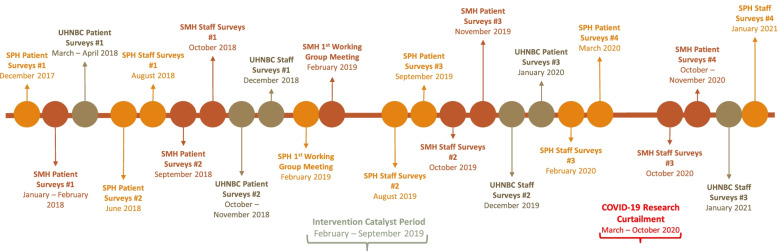
Patient and staff survey data collection and intervention timeline

We hypothesized the percentage of patients who leave without care being completed, and staff sick time as a proportion of productive hours would decrease post-intervention. For both primary and secondary outcomes, we hypothesized that there would be a significant change immediately after the intervention with a continued improving trend. Qualitative data in the form of patient comments regarding care and the reasons they felt they experienced discrimination in the ED were used in conjunction with other survey data to describe patient experiences and have been reported previously [43]. Qualitative data were used to describe impacts of participation on staff, leadership and the organizational culture, 

Data were collected pre-intervention, including two waves of patient survey data, one wave of staff survey data and administrative data over the time period January 2017 – December 2020. As shown in Fig. 1, patient survey data collection commenced in December 2017, was curtailed by COVID 19 restrictions between March–October 2020, and halted as of November 2020.

This paper reports on the outcomes of longitudinal analysis of patient and staff survey responses, and administrative data. Staff surveys were administered at the same time as Wave 2 (pre-intervention) and Waves 3 and 4 of patient data collection. Despite an initial staff survey sample of *n* = 393 at Wave 1 across the three sites, sample sizes for the second (*n* = 109) and third (*n* = 131) waves were too small to analyze longitudinally. Thus, we were unable to test our hypotheses related to changes in staff perceptions of care, staff engagement and team effectiveness.

### Power analysis

The power analysis was based on the within-site analyses. For the longitudinal patient survey data, we estimated the number of patients needed per site to detect a small effect size of 0.25. We calculated the means and standard deviations from the EQUIP PHC study data for patients’ experiences of care to inform the power analysis. With power of 0.80 and alpha of 0.05, we could detect significant changes with an effect size of 0.25 with a sample size of 250 patients at each site at each wave. We used methods suggested by Zhang et al. [63] to estimate the power for the interrupted time series (ITS) design. Based on the trend over time in the primary ITS outcomes extracted from the administrative data for the past 24 months, we estimated that the time series would have an autocorrelation of − 0.20 and would require an autoregressive model with 1 lag (AR1). Power is 0.85 to detect a moderate effect size with alpha of 0.05, AR1 model, and an autocorrelation of − 0.20 with 24 time points prior and 12 time points post the intervention.

### Measures

As outlined in the protocol [42] and shown in Tables 1 and 5, patients were surveyed regarding their demographic characteristics, their experiences of discrimination in the ED, and their overall ratings of care during their ED experiences.

### Data collection

Every consecutive patient presenting to the EDs who was over the age of 18 and appeared able to consent (e.g. was conscious) was approached to participate (*n* = 4771); these data were collected in 4 waves at two sites and 3 waves (due to pandemic constraints) at the third site, with 2 waves pre-intervention at all sites (See Table 4).

**Table 4:** Participants consenting and completing by wave and site

Site	Wave 1		Wave 2		Wave 3		Wave 4		Total	
	Part 1	Part 2	Part 1	Part 2	Part 1	Part 2	Part 1	Part 2	Part 1	Part 2
SPH	229	155(68%)	477	327(69%)	491	359(73%)	464	308(66%)	1661	1149(69%)
SMH	422	259(61%)	520	405(80%)	480	341(71%)	433	284(66%)	1855	1289(69%)
UHNBC	289	212(73%)	487	334(69%)	465	324 (70%)	NA	NA	1241	870(70%)
TOTAL	940	626(67%)	1484	1066(72%)	1436	1024(71%)	897	592(66%)	4771	3315(70%)

Data were collected at all times of the day and all days of the week. Patients who were not able to communicate in English were provided with information in their language of choice (written materials, including consent forms, were available in Hindi, Punjabi, and Traditional Chinese,^1^ and translators were available). We approached as many patients as possible during each data collection shift, estimating we approached 80% of patients; approximately 50% of those approached consented to participate. Patients were consented and enrolled at any time during their ED visit or at the conclusion of their visit. Demographic and contact information were collected on enrollment (Part 1), with remaining data (Part 2) collected at the conclusion of their visit (after discharge, transfer or admission to hospital). A final sample of 3315 answered post-visit questions (both Part 1 & 2). Data were collected in person or by phone follow-up directly on tablets by trained research staff. Further details of data collection are reported elsewhere [43]. The rate between enrollment and completion was 70%. Other than the first wave at the first site, the sample sizes all exceeded the sample size required for the power analysis.

We collected administrative data retrospectively at equally spaced intervals (monthly) for 24 months prior to the start of the intervention, 12 months during the implementation of the intervention and 12 months post-intervention for each variable. Because the data collection and intervention activities were staggered, these data were collected for 4 years at each site for a different range of dates (January 2017 – December 2020).

### Analysis

Longitudinal analyses of patient survey data were conducted within site. For each wave of patient and staff survey data, descriptive statistics appropriate to the level of measurement were computed. Since data were collected from different patients at each wave, analysis of variance with time as the independent variable was used to examine change in quality of care across time. Chi-square was used to examine perceived discrimination by time. To examine patient characteristics associated with perceived quality of care linear regressions were used. Separate models for Indigenous identity, employment status, income difficulty, and age > 65 were tested. As noted, sample sizes for the staff survey precluded longitudinal analysis. Descriptive analysis of the first wave of staff data will be reported elsewhere.

Administrative data were analyzed using segmented regression with autoregressive models [66, 67]. We compared the level of the outcome (% patients LWCC) and the rate of change over time between the time period prior to the intervention and the time period after the intervention [67]. We estimated the level and slope across time of the outcomes prior to the intervention and changes in the level and slope after the intervention. The change in level provides an estimate of the immediate effect of the intervention, and the change in slope provides an estimate of the ongoing effect of the intervention across time after implementation.

## Results

The sample of patients was highly diverse in terms of social location, and was generally representative of the underlying populations served by each ED. In comparison to the underlying provincial population, our sample had greater representation from people over 65, people accessing homelessness shelters, and Indigenous people (see Table 5).

**Table 5:** Demographic characteristics of patients completing (*N* = 3315)

Variable	n (%) of EQUIP ED Sample	n (%) of BC EDPEC Sample	n (%) of BC Census Sample
**Canadian Triage & Acuity Scale (CTAS)**			NA
1 - Resuscitation	11 (0.5)	39 (0.3)	
2 - Emergent	434 (20.4)	2018 (16)	
3 - Urgent	1022 (48.0)	5789 (45.9)	
4 – Less urgent	601 (28.2)	4023 (31.9)	
5 – Non-urgent	61 (2.9)	580 (4.6)	
**Age**	Range: 18–99, Mean: 50.8, SD: 18.610	NA	Range: 0–100+, Mean: 42.3, Median: 43.0 [64]
**Age 65 and over**			
Under 65	2436 (74.7)	9530 (67.6)	3,799,070 (81.7)
Over 65	826 (25.3)	4546 (32.4)	848,985 (18.3)
**Gender**			
Woman	1629 (49.4)	7568 (53.9)	2,369,815 (51.0)
Man	1635 (49.6)	6506 (46.1)	2,278,245 (49.0)
Non-binary	34 (0.9)	1 (0)	NA
**Education**			
Didn’t complete secondary school / high school	650 (19.8)	4341 (29.8)	601,640 (15.5)
Completed secondary school / high school	704 (21.5)	2835 (19.8)	1,138,565 (29.4)
Some or completed post-secondary	1922 (58.7)	5972 (46.2)	2,130,175 (55.0)
**Born in Canada**		NA	
No	907 (27.4)		1,292,675 (30.5)
Yes	2379 (72.4)		3,167,155 (69.5)
**First language English**		NA	
No	789 (28.7)		1,428,305 (31.1)
Yes	1656 (71.3)		3,170,110 (68.9)
**Speaks English**		NA	
Does not currently speak English	87 (2.6)		151,760 (3.4)
Currently speaks English	3210 (97.4)		4,442,695 (96.6)
**Indigenous**			
Non-Indigenous	2720 (82.9)	12,116 (94.1)	4,289,655 (94.1)
Indigenous	560 (17.1)	1246 (5.9)	270,585 (5.9)
**Living situation – dichotomized**			NA
Precarious housing^a^	383 (11.6)		
Stable housing	2909 (88.4)		
**Accessed a shelter in the past year**		NA	NA
No	3036 (92.4)		
Yes	250 (7.6)		
**Primary work status**		NA	
Employed FT or PT	1464 (44.7)		2,305,690 (59.6)
Unemployed	760 (23.2)		165,975 (4.3)
Retired	838 (25.6)		1,398,710 (36.1)
Other (includes seasonal, exchange services or student)	213 (6.5)		
**Receiving social assistance**^b^		NA	
Not receiving	2415 (88.6)		4,073,315 (98.4) [65]
Receiving	312 (11.4)		67,821 (1.6)
**Receiving disability benefits**		NA	NA
Not receiving	2122 (76.0)		
Receiving	669 (24.0)		

^a^The response options included in “precarious housing” are: couch-surfing, shelter, on the street, in vehicle (car or van), SRO, rooming house, RV or trailer, Tent, and other

^b^In BC, a single person on income assistance receives $935 each month, while a single person on disability assistance receives $1358.42

Compared to a sample obtained from Emergency patients in the same province during a similar time frame using mail out surveys (Table 5 with comparisons to British Columbia Emergency Department Patient Experiences of Care - BCEDPEC), our sample tended to include more adults under the age of 65 and be more highly educated, with poorer self-reported health and higher acuity. Similar to the findings by Chiu et al. [68], which compared the BCEDPEC sample to a sample collected in-person, our sample was more diverse in terms of having greater representation from Indigenous people and those less likely to have a usual primary care home (meaning access to an identified physician or nurse practitioner as their regular health care provider). Additionally, as reported elsewhere [43], our pre-intervention sample was more diverse, particularly on demographic variables indicating structural disadvantage, than samples collected routinely by mail out surveys.

Across all waves, comparisons between those consenting and enrolling, and those completing showed that those who completed were less likely to identify as Indigenous, have precarious housing, or have accessed a shelter in the past 6 months, signaling a less disadvantaged final sample than those presenting to the EDs. They were also more likely to be over age 65; given that our prior analysis [43] showed intersections among being over 65, low financial strain and stable housing, this may also signal that those completing the full survey experienced less structural disadvantage than the overall population presenting for care.

### Site specific analyses

Analysis of variance showed encouraging trends in the patient outcomes: patient ratings of quality of care (primary) and patient experiences of discrimination in the ED (secondary). As shown in Table 6, at UHNBC (no intervention), patient perceptions of quality of care were significantly lower between W1 and W3 (*p* = .020). These changes were accompanied by a change in demographics from W1 and W2, in that the sample was younger and more participants identified as Indigenous. The change over time in quality of care is no longer significant after adjusting for age and identifying as Indigenous (*p* = 0.097).

**Table 6:** Perception of quality of care and discrimination over time

Site	Wave 1	Wave 2	Wave 3	Wave 4	*p*-value
Perceived Quality of Care Mean (SD)					
SPH	8.32 (1.83)	8.64 (1.47)	8.21 (2.07)	8.23 (2.09)	0.013
SMH	7.80 (2.24)	8.21 (1.91)	8.07 (2.17)	8.11 (2.14)	0.111
UHNBC	8.83 (1.62)	8.51 (1.86)	8.38 (1.91)	N/A	0.020
Any Experience of Discrimination Percentage					
SPH	25.2	17.3	26.9	27.0	.081
SMH	28.1	23.4	28.9	21.9	.118
UNHBC	16.2	19.9	18.6	N/A	.553

At SMH (some intervention activity) there was no change in patient perceptions of quality of care over time. Demographics were stable over time except for age (there were more people over 65 at W2 and W3 compared to W1 and W4).

At SPH, where the most sustained intervention activities were enacted, patient’s perceptions of quality of care were significantly higher at W2 compared to W3 and W4 (*p* = .013). However, when controlling for changing patient characteristics, the differences in quality of care were no longer significant (*p* = .212). Changes in perceived quality of care were explained by changing demographics at Wave 2, when the sample was more likely to be born in Canada, have English as a first language, have less financial strain, and less likely to be: Indigenous, unemployed, or to have accessed a shelter in the last 6 months.

Variability over time in relation to demographic changes suggested a supplementary analysis to identify ‘who’ experienced lower quality of care. This analysis showed that specific groups of patients experienced different ways at each site. At UHNBC people who identified as Indigenous (β = − 0.13, *p* < .001), who were unemployed (β = − 0.16, p < .001), younger (β = 0.20, p < .001) and those experiencing financial strain (b = − 0.13, p < .001) reported lower perceptions of quality of care at all time points. The same associations were found for SPH - Indigenous (β = − 0.10, *p* = .001), unemployed (β = − 0.20, p < .001), younger (β = 0.13, p < .001) and those experiencing financial strain (b = − 0.19, p < .001). At SMH, younger (b = 0.16, p < .001), unemployed (b = − 0.12, p < .001) and those experiencing financial strain (b = − 0.16, p < .001) reported receiving lower quality of care.

**Fig. 2:**
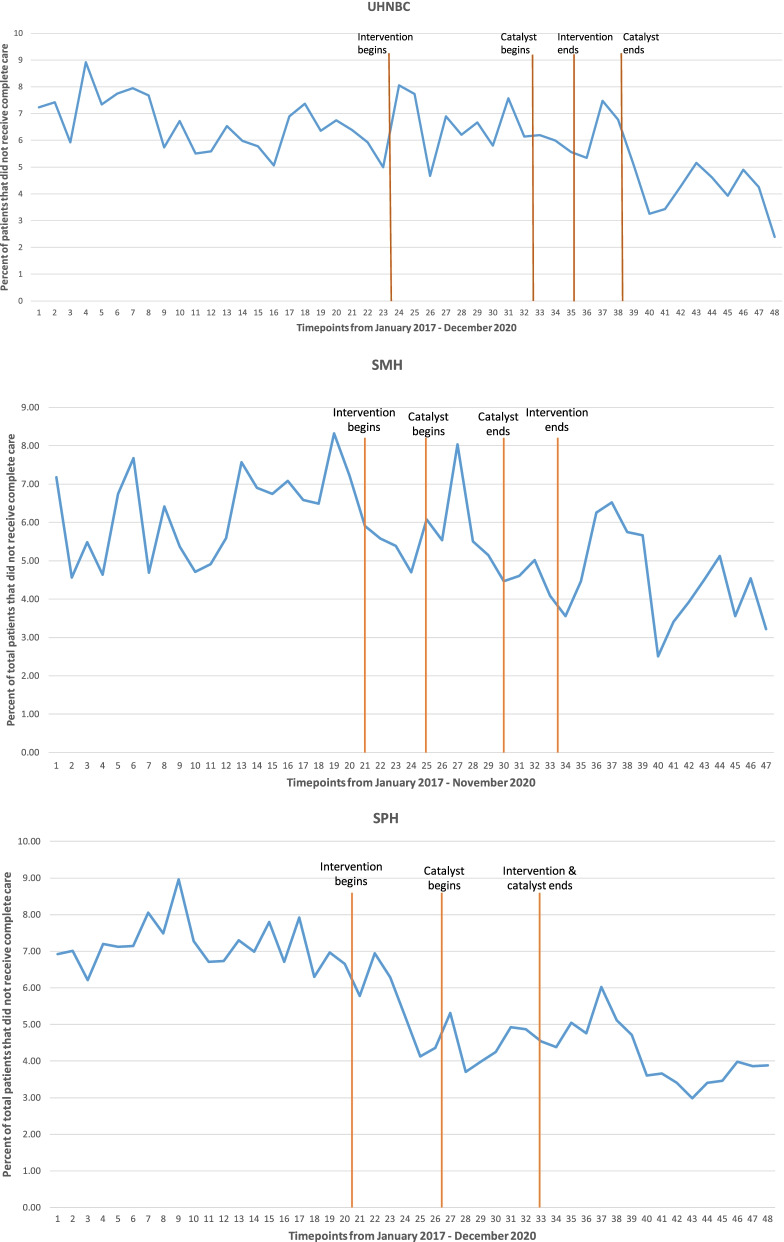
ITS analysis for LWCC for all three sites

As shown in Fig. 2, analysis of administrative data regarding the percentage of people leaving without care completed showed that whereas there were no changes at SMH or UHNBC, at SPH there was a significant decrease in the percentage of patients who leave without care being completed from the pre- to post implementation period (b = − 1.49, p = .001) and a significant improvement in the trend over time in the post-intervention period compared to the pre-intervention period (b = − 0.069, *p* = .038). There were no differences in staff sick time from pre- to post intervention at any of the three sites.

## Discussion

This analysis offers new insights about what is required to promote equity at an organizational level in EDs. Most research on improving equity in health care aims at training and educating individual health care providers on topics such as implicit bias, Equity, Diversity, Inclusion (EDI), cultural competence, the social determinants of health, how to respond to violence and aggression, or trauma-informed practice [15–19, 69–72], and often, focuses improving outcomes for particular ethnocultural groups (defined on the basis of singular categories, variables of group affiliations) [73, 74]. While research shows that providing staff with such education may be necessary, it is insufficient to address the organizational and individual level gaps in providing non-judgmental, culturally safe care, leading to calls for wider organizational and policy interventions (e.g. [75]). Further, education must be embedded as part of broader organizational and system transformation [76, 77]. Stated otherwise, training and educational interventions alone cannot enhance the equity-orientation of organizations, and shifting knowledge, attitudes, and behaviours of individuals is not sustainable without organizational supports, policy directives, accountability mechanisms, and whole-organization actions [76]. There is, however, little guidance as to how to affect change beyond increased awareness and skill-building at the level of individual staff members. The Equipping Health Care for Equity intervention study is one of the first efforts we know of to seek to create change in support of EOHC in EDs at an organizational level.

This study was conducted in the context of two significant health and health care crises affecting Canada during the study timeframe: the crisis in drug toxicity-related overdoses and deaths, and the COVID 19 pandemic, with the latter also exacerbating the catastrophic impacts of the former [78]. The intervention activities were shaped by the unique contexts of each ED, and by their role in relation to responding to the drug toxicity crisis. In each of their respective health authorities, the three participating EDs are the largest EDs in their catchment and serve as referral centers for people who use drugs; thus, they provided care to the majority of people who experienced overdose and those who died. Provincial data illustrates that the Northern Health Authority within which UHNBC is located, had a much higher rate of deaths from overdose [79]. This both contributed to the capacity of staff to engage with an understanding of EOHC that explicitly foregrounds harm reduction, and shaped the ways staff engaged with the intervention. For example, at SMH there was a project on harm reduction that ran concurrent with the EQUIP intervention period; later, a large proportion of the nursing leadership and staff from the ED were redeployed to the pandemic response. COVID responses ultimately halted intervention and data collection activities at all sites.

Overall, the intervention activities were modest at two of the EDs, and were not initiated at the third. At both SMH and SPH, the activities were mostly confined to a small group of ED nurses, with little engagement beyond the working group. The lack of engagement of staff beyond the WGs can also be seen in the low response rate to staff surveys beyond the initial survey. Thus, the intervention activities may not have been sufficient to affect change, particularly within the short timeframe that we intentionally imposed based on our research in primary care settings. The “dose” of intervention required to affect measurable change toward equity requires further study – the right combination of momentum and the time frame required may be quite contextually and situationally dependent. The changing context may have negatively impacted care at all sites; however, no decrease was seen in patients’ overall ratings of care at SPH and SMH where intervention activities were undertaken, whereas a significant decrease in ratings of care was seen at UHNBC, albeit explained by the fact that more people who tend to report poorer care (younger and Indigenous people) were sampled. It maybe that the intervention activities at SPH and SMH contributed to offsetting the negative impacts of a worsening care context. Further, the direction of non-significant improvements suggests potential for further change with more fulsome activity. At SPH, where we saw the only significant improvement in outcome, the EQUIP intervention aligned with the commitment to equity as evidenced by the other equity-oriented initiatives being undertaken and the resources available (e.g. a full staff complement, a person to facilitate setting up meetings, complementarity to the Safe Care program simultaneously initiated). At UHNBC, the importance of equity was also well recognized and discussed among leadership in the ED, and by some direct care staff physicians, but the resources and pressures were such that it was not until a subsequent provincial report on an investigation into Indigenous-specific racism in health care was released [80] - after the EQUIP intervention period - that dedicated internal resources, such as people to coordinate meetings, book rooms, etc. and leadership were committed to examine activities that could support cultural safety within the ED.

Importantly, the observation of a significant decrease in the percentage of patients who leave without care completed at SPH was especially encouraging, because it was the site with the most enduring intervention activity. Calls for improving care and equity-oriented care in EDs highlight the need to identify those with the highest levels of unmet needs and strategies to address those needs [81, 82]. Research suggests that the characteristics of those who leave without care completed (LWCC) include those who have the highest levels of unmet health care needs. Patient populations overrepresented among those who LWCC include those with mental health issues/psychiatric needs [83, 84], substance use health problems [82, 83, 85], chronic pain [7], unstable/low income housing [82, 85] and frequent use of the ED [84, 85]. Some authors suggest that this overrepresentation is related to unmet needs for these populations and that additional associated resources would decrease the rate of LWCC [82, 83], such as having dedicated social workers in the ED [82] or increased access to mental health resources [83]. For example, Doupe et al. [82] studied 122, 639 patients in Manitoba with 250,754 ED visits, of whom 2.3% made 3387 visits they termed as “paradoxical” in that the patients arrived by ambulance but left without seeing a care provider. They found that those with paradoxical visits were frequent ED users, lived in the lowest income areas, used substances and had high levels of primary care physician visits. Efforts to reduce the number of people who leave without care completed may contribute to lessening both unmet health care needs, readmission rates, and frequent ED use.

The study was limited by the chronic, underlying staffing shortages, especially of nurses, at UHNBC, and given that it was primarily nurses leading the intervention work, this made it impossible for a working group to form. Indeed, the working group that has formed post-intervention is being led by physicians and two Indigenous Elders all of whom are external to the Health Authority. Relatedly, the study was limited by the lack of interprofessional engagement, which we identified in EQUIP PHC as critical to fostering organizational-cultural change toward EOHC [29, 30]. The two working groups were comprised primarily of nurses, with some initial involvement from physicians (SPH), security personnel, care aids and an Indigenous liaison (SMH).

Methodologically, because we used a longitudinal panel design we had to take changing demographics into account at each wave. However, a cohort study would not have been possible, given the unpredictable patterns of ED visits; while we potentially could have followed repeat users of EDs, this would not have answered our research questions or provided a basis for comparisons with the wider ED patient population. The limitations of the measures used have been described elsewhere [43], except for the administrative data, which was itself limited in several ways. First, because each ED is within a different health authority, data were collected differently between settings. Further, staff data and patient data were collected using systems unique to each setting, meaning there were three sets of staff data and three sets of patient data. We requested and obtained all available staff and patient variables at each setting, and while there were other candidate variables that may have been equity sensitive (e.g., measures of violence and aggression), few were comparable across settings. Only the number of patients who left without care being completed and staff sick time met the criteria of being potentially equity-sensitive, comparable among sites and not explained by other influences (e.g., changes in staff stress-leaves/medical-leaves could not be attributed to the intervention due to multiple influences). Further, because we were not able to collect data on presenting diagnoses, we were not able to study primary care sensitive conditions [86, 87], which may have permitted a more nuanced analysis of those leaving without care completed. Finally, it was not possible within the administrative data to link variables indicating structural disadvantage (e.g. homelessness, readmissions) so we were unable to do subgroup analyses on these data.

## Conclusions

This study showed some encouraging trends in the impact of efforts to promote equity-oriented care. The trends in patient ratings of care and the significant decrease in the percentage of patients who leave without care being completed suggest potential for significant change. However, changes over time cannot be attributed to success or failure of intervention activities alone. Rather, because movement toward equity will occur within a wider context, alignment should be sought with other initiatives, as was clearly the case at SPH during the intervention timeframe, and after the EQUIP study period at all three sites. While the intervention period ended and data collection was completed in 2020, UHNBC began intervention work in the fall of 2021, drawing on the catalyst grant provided by the study; in the spring of 2022, SPH began follow up work focused on using an equity approach to decreasing violence and aggression in the ED; also in the spring of 2022, SMH restarted initiatives begun by their working group, requesting an extension to spend their catalyst grant. This suggests that while a research study may offer an initial start to explicit equity work, the actual implementation is a long term, and likely unending effort.

Importantly, we think that “whole of hospital”, “whole of community” and “whole system” approaches hold promise. The experience of UHNBC suggests that EDs in smaller settings are unlikely to have the resources to mount equity-oriented changes without the involvement of other departments both within the hospital and community. Our challenges in comparing data across sites suggests that better alignment of administrative data collection across sites would allow better measurement and monitoring.

We underestimated the support that direct care staff required to organize a working group, and recommend more direction regarding how to do so, and more concrete support, especially initially. Further, although educational opportunities were offered, uptake of the offer was minimal, and mostly confined to the Working Group members, with a consequence that few staff were exposed to the basics of equity. Thus, we recommend that a minimum level of staff training/education on EOHC approaches be provided followed by opportunities for interdisciplinary discussions of the implications of EOHC, and opportunities for brainstorming more impactful and disruptive intervention activities. These kinds of intervention activities will require integration and involvement of management and executive leadership more directly, greater interprofessional engagement, and broader uptake among staff. From our analysis of interviews with leaders and working group members , we recommend comprehensive planning involving both leaders and direct care staff to assess and deepen preparedness for change and to build momentum. To aid organizations to enact these recommendations, we have created an EQUIP Equity Action Kit aligned with the Active Implementation Frameworks (AIFs) [88] providing guidance and resources to support each step, including staff education tools ranging from brief text and video “essentials” that take less than 10 minutes to view, to longer on-line learning modules, and a series of action tools, all available at https://equiphealthcare.ca/.

## Data Availability

The datasets generated and/or analysed during the current study are not publicly available because we are still actively working on analyses, but are available from the corresponding author on reasonable request.

## Abbreviations

BCEDPEC: British Columbia Emergency Department Patient Experiences of Care
EOHC: Equity-Oriented Health Care
EQUIP: PHC EQUIP Primary Health Care
EDI: Equity, Diversity, Inclusion
FLO: Front-Line Ownership
ITS: Interrupted Time Series
LWCC: Left Without Care Complete
PHC: Primary Health Care
SMH: Surrey Memorial Hospital
SPH: St. Paul’s Hospital
UHNBC: University Hospital of Northern British Columbia
WGs: Working Groups

## Footnotes

1 Cantonese and Mandarin are spoken dialects of Chinese, not written formats. The written format can use either Traditional or Simplified Chinese. The translation firms and our consultants suggested we translate materials to Traditional Chinese to cover both Cantonese and Mandarin speakers.
